# Influence of Solid Drug Delivery System Formulation on Poorly Water-Soluble Drug Dissolution and Permeability

**DOI:** 10.3390/molecules200814684

**Published:** 2015-08-13

**Authors:** Marko Krstić, Miljana Popović, Vladimir Dobričić, Svetlana Ibrić

**Affiliations:** 1 Department of Pharmaceutical Technology and Cosmetology, Faculty of Pharmacy, University of Belgrade, Vojvode Stepe 450, P.O. Box 146, Belgrade 11221, Serbia; E-Mails: mkrstic109@gmail.com (M.K.); miljana.popovic17@gmail.com (M.P.); 2 Department of Pharmaceutical Chemistry, Faculty of Pharmacy, University of Belgrade, Vojvode Stepe 450, P.O. Box 146, Belgrade 11221, Serbia; E-Mail: vladimir@pharmacy.bg.ac.rs

**Keywords:** carbamazepine, dissolution rate, PAMPA test, solid dispersions, S-SMEDDS, S-SNEDDS, characterization

## Abstract

The majority of drugs have a low dissolution rate, which is a limiting step for their absorption. In this manuscript, solid dispersions (SD), solid self-microemulsifying drug delivery systems (S-SMEDDS) and solid self-nanoemulsifying drug delivery systems (S-SNEDDS) were evaluated as potential formulation strategies to increase the dissolution rate of carbamazepine. Influence of increased dissolution rate on permeability of carbamazepine was evaluated using PAMPA test. In S-SMEDDS and S-SNEDDS formulations, the ratio of liquid SMEDDS/SNEDDS and solid carrier (Neusilin^®^ UFL2) was varied, and carbamazepine content was constant. In SD formulations, the ratio of carbamazepine and Neusilin^®^ UFL2, was varied. Formulations that showed the best dissolution rate of carbamazepine (SD_1:6, SMEDDS_1:1, SNEDDS_1:6) were mutually compared, characterization of these formulations was performed by DSC, PXRD and FT-IR analyses, and a PAMPA test was done. All formulations have shown a significant increase in dissolution rate compared to pure carbamazepine and immediate-release carbamazepine tablets. Formulation S-SMEDDS_1:1 showed the fastest release rate and permeability of carbamazepine. DSC, PXRD and FT-IR analyses confirmed that in S-SMEDDS and S-SNEDDS carbamazepine remained in polymorph form III, and that it was converted to an amorphous state in SD formulations. All formulations showed increased permeability of carbamazepine, compared to pure carbamazepine.

## 1. Introduction

Oral administration of drugs is considered to be the most natural, uncomplicated, convenient and safe method. Since nearly one third of drugs are poorly water soluble, oral bioavailability of those drugs could be an issue [1]. Carbamazepine is an antiepileptic agent with low solubility (0.15 ± 0.07 mg/mL, 25 °C, [2]) and high permeability, which makes it a great candidate for this study. Also, carbamazepine is prescribed in high doses, so this also represents a challenge for researchers. According to Biopharmaceutical Classification System (BCS) that classifies drugs into four categories according to their permeability and solubility properties, carbamazepine is classified as a class II drug. As dissolution of poorly water-soluble drugs (e.g., carbamazepine) is the rate-limiting step for absorption, improvement of dissolution rate should increase drug absorption and bioavailability [3,4]. Other than that, carbamazepine is susceptible to polymorphic transition, so it is a great candidate in the evaluation of the influence of excipients on polymorphic form.

Various techniques have been introduced in order to enhance the dissolution rate and solubility of drugs, such as physical (particle size reduction, drug dispersion in carriers, complexation and solubilization by surfactants), chemical and other methods [1,5]. Various methods for enhancing the solubility of carbamazepine were evaluated: dispersion of carbamazepine in carriers [3,6,7,8], complexation [9] and solubilization by surfactants [2,10,11,12].

Solid dispersions are molecular mixtures of poorly water-soluble drugs in hydrophilic carriers, where the drug release profile is driven by polymer properties. Management of the drug release profile using solid dispersions is achieved by manipulation of the carrier and solid dispersion particle properties. Parameters, such as carrier molecular weight and composition, drug crystallinity and particle porosity and wettability, when successfully controlled, can produce improvements in bioavailability [13].

Self-nanoemulsifying drug delivery systems (SNEEDS) are isotropic mixtures of oil, surfactant (HLB > 12) and co-surfactant. These systems are spontaneously emulsified when exposed to GIT fluids and form oil-in-water nanoemulsions. The droplet size of oil in these nanoemulsions is 100–250 nm [14,15].

Self-microemulsifying drug delivery systems (SMEDDS) are isotropic mixtures of oil, surfactant (HLB > 12) and co-surfactant. In comparison with SNEDDS, the content of hydrophilic surfactants and cosurfactants is increased and lipid content is reduced. The risk of drug precipitation in SMEDDS is higher due to lower lipid content [16]. Under the influence of GIT movements they form a fine oil-in-water emulsion. After the dispersion in aqueous media, these systems form homogeneous, transparent, isotropic and thermodynamically stable microemulsions [17]. The droplet size of oil in these microemulsions is less than 100 nm [15].

However, self-emulsifying formulations are normally prepared as liquids that have some disadvantages, for example, high production costs, low stability and portability, low drug loading and different dosage forms. Irreversible drugs/excipients precipitation may also be problematic. More importantly, the large quantity of surfactants in the formulations can induce gastrointestinal irritation. In order to avoid these problems, solid self-emulsifying drug delivery systems (SSEDDS) have been investigated as alternative formulations. Solid self-emulsifying drug delivery systems combine the advantages of liquid SEEDS (*i.e.*, enhanced solubility and bioavailability) with those of solid dosage forms (e.g., low production cost, convenience of process control, high stability and reproducibility, better patient compliance) [18].

Dissolution and membrane permeability of drugs through the mucosa are the key parameters in the gastrointestinal absorption. [4]. While a significant increase of apparent solubility may be achieved by some solubility-enabling formulations, the impact of these formulations on permeability of a lipophilic drug is often overlooked. Use of surfactants as a strategy to increase the apparent aqueous solubility of lipophilic drugs may lead to increased, decreased or unchanged membrane permeability. Micellar solubilization of drugs allows extraordinary increases of solubility, but also results in a decreased free fraction of drug available for intestinal membrane permeation. Consequently, permeability decreases with the increase of surfactant concentration above the critical micelle concentration (CMC). A direct tradeoff exists between the apparent solubility and permeability irrespectively of the free fraction, which must be taken into account when developing solubility-improving formulations in order to maximize the overall oral absorption [19].

Several *in vitro* methods for the intestinal absorption assessment have been developed. These methods include Caco-2 and other “intestinal-like” cells (MDCK, TC-7, HT29-MTX, 2/4/A1, T-84, LLC-PK1, A549…), Everted sac and Ussing chamber, *in-situ* rat intestinal perfusion, artificial membrane and Follicle-Associated Epithelium Model (FAE) [20,21].

The artificial membrane is used as an alternative model for GI membrane in the assessment of drug absorption potential. A majority of drugs are absorbed primarily or partially through passive transport. Therefore, the rate of permeation through a simple artificial membrane, which mimics passive transcellular transport, provides a good indication of a drug’s absorption potential [22].

The most used *in vitro* methods for prediction of permeability are cell-based (especially Caco-2 cells) and PAMPA (parallel artificial membrane permeability assay). PAMPA is considered a quick and simple test that measures passive permeability in the absence of efflux systems or transporters [21]. Since carbamazepine is a substance that is absorbed by passive transcellular transport, PAMPA test is a convenient method for evaluation of its permeability.

The aim of this study was to investigate the possibility of increasing the dissolution rate of carbamazepine by formulation of different types of solubility-enabling formulations. Using carbamazepine as a model substance, three different types of formulations were prepared: solid dispersions, self-microemulsifying drug delivery systems (SMEDDS) and self-nanoemulsifying drug delivery systems (SNEDDS). Additionally, the aim was to evaluate the influence of increased solubility on permeability of carbamazepine, using PAMPA test.

## 2. Results and Discussion

### 2.1. Droplet Size Analysis

**Figure 1:**
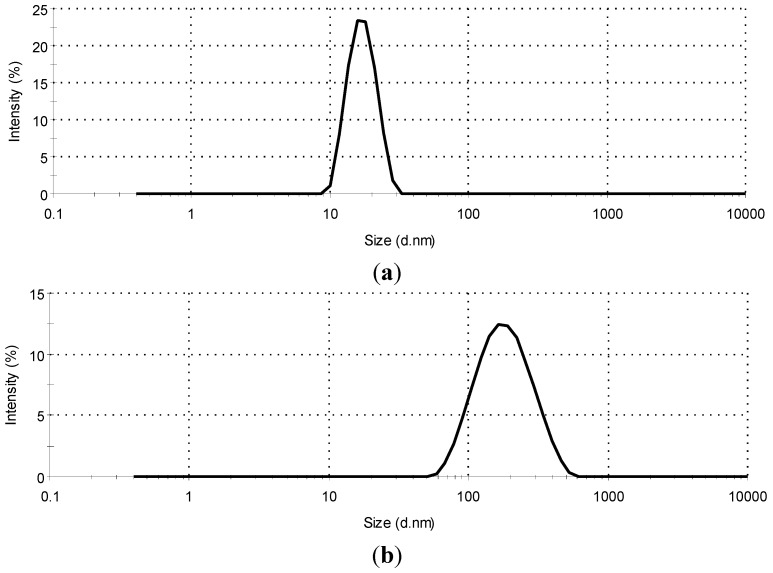
The droplet size distribution by intensity for SMEDDS (self-microemulsifying drug delivery systems) (**a**) and SNEDDS (self-nanoemulsifying drug delivery systems) (**b**) emulsions after dilution with water phase up to 90% *w*/*w*.

The droplet size of SMEDDS and SNEDDS without carbamazepine was analyzed. It can be concluded that upon high water dilution, SMEDDS is capable of forming oil-in-water microemulsions, because the droplet size is less than 100 nm. Polydispersity is the ratio of standard deviation to mean droplet size and indicates the uniformity of droplet size within the formulation. It is a general rule that the higher the polydispersity, the lower the uniformity of the droplet size in the formulation [23]. SMEDDS had monomodal droplet size distribution with a PDI value of 0.104 ± 0.005 and droplet size of (17.66 ± 0.17 nm), which indicates that a stable microemulsion was formed. SNEDDS had a monomodal droplet size distribution with a PDI value of 0.184 ± 0.012 and a droplet size of 156.3 ± 0.28 nm, which indicates that a stable nanoemulsion was formed (Figure 1).

### 2.2. Fourier-Transform Infrared Spectroscopy (FT-IR)

**Figure 2:**
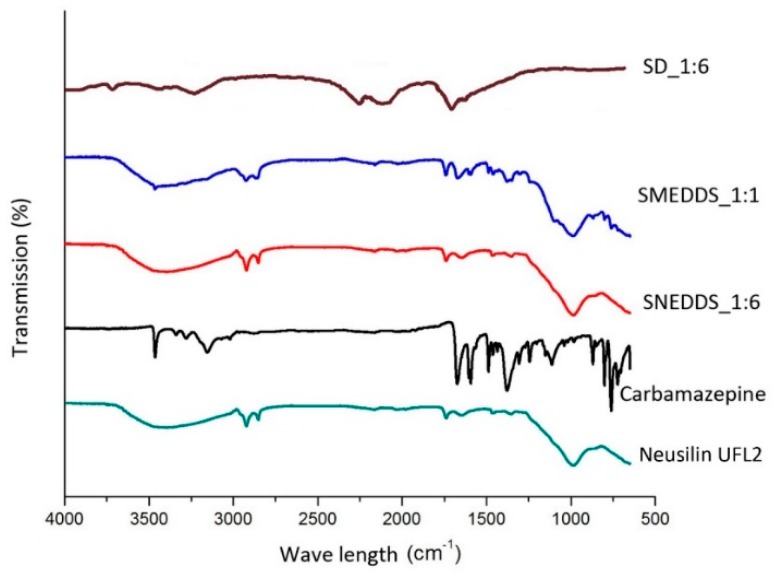
FT-IR spectra of evaluated formulations and pure carbamazepine.

FT-IR analysis of selected formulations and pure carbamazepine were performed in order to characterize the physical state of carbamazepine and to identify potential interactions between carbamazepine and Neusilin^®^ UFL2. The FT-IR spectra of carbamazepine showed a characteristic peak at 3462 cm^−1^ (–NH valence vibration), 1676 cm^−1^ (–CO–R vibration), 1598 cm^−1^ (–C=C– and –C=O vibration) and 1384 cm^−1^ (–NH deformation) [24]. Spectrum of SD_1:6 shows significant diminution of characteristic peaks in fingerprint region, which indicates presence of interactions between carbamazepine and Neusilin^®^ UFL2. Peak of NH vibration is present at 3462 cm^−1^, but it is blunt and diminished. Other three characteristic peaks are also present, but not very prominent. FT-IR spectra of S-SMEDDS_1:1 and S-SNEDDS_1:6 showed all characteristic peaks for carbamazepine, indicating that no interactions occurred between carbamazepine and solid carrier, so it can be concluded that in these formulations carbamazepine stayed in polymorph form III. The line intensity is slightly weaker, which is expected, due to the smaller share of carbamazepine in formulations. FT-IR spectra of evaluated formulations are shown in Figure 2.

### 2.3. Differential Scanning Calorimetry (DSC)

**Figure 3:**
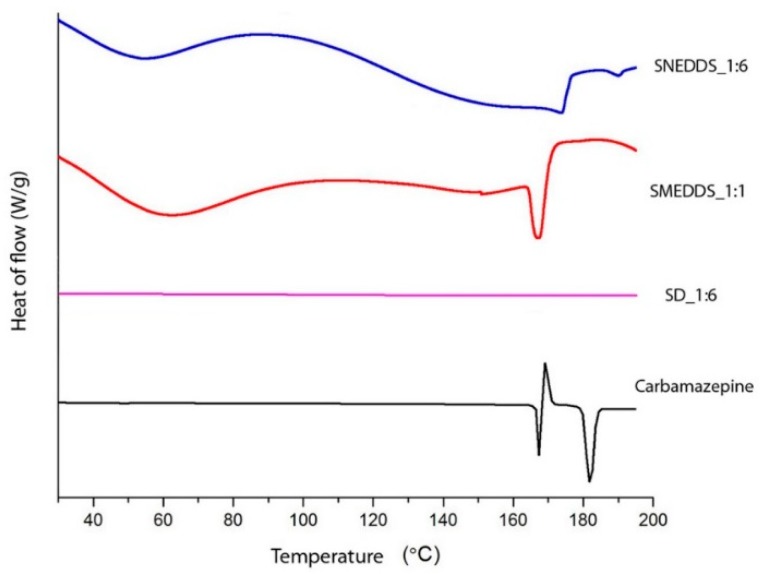
DSC curves of evaluated formulations and pure carbamazepine.

DSC curves of pure carbamazepine and tested formulations are shown in Figure 3. During heating, the sample of pure carbamazepine melts, which corresponds to the first endothermic peak at 175.08 °C, contributing to melting of polymorph form III, after which the exothermic peak is observed at 176.63 °C, which comes from the recrystallization of polymorph form III to polymorph form I. Another endothermic peak is observed at 190.95 °C, which comes from melting the polymorph form I of carbamazepine. These results are in accordance with our previous results and results obtained from Grzesiak *et al.* [25]. In SMEDDS_1:1 and SNEDDS_1:6 formulations, characteristic peaks of melting of carbamazepine polymorph form III were observed at the temperature range of 150–175.8 °C, indicating that in these formulations carbamazepine probably has not changed its polymorph form. In SD_1:6 formulation, this peak is not noticed, which indicates that in this formulation carbamazepine was probably converted into an amorphous form, which will be confirmed later by PXRD.

### 2.4. Powder X-ray Diffraction Analysis (PXRD)

**Figure 4:**
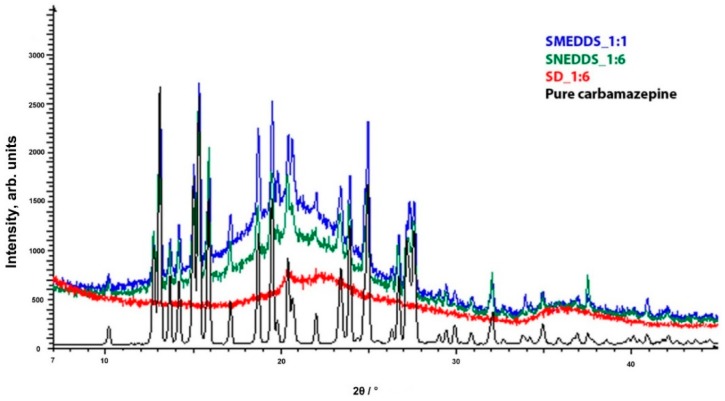
PXRD patterns of evaluated formulations and pure carbamazepine.

Figure 4 illustrates the PXRD patterns of pure CBZ and the optimal formulation. The PXRD pattern of CBZ exhibits characteristic high-intensity diffraction peaks at 13.02°, 15.22°, 15.78°, 19.40°, 24.92°, 27.50° and 31.86 2θ, which is in accordance with diffractograms previously reported for crystal form III [25,26]. This characteristic peaks are observed in S-SMEDDS_1:1 and S-SNEDDS_1:6, which confirms assumptions given by DSC and FT-IR analyses that carbamazepine remains in polymorph form III. In the diffractogram of SD_1:6, no peaks are observed, which indicates that carbamazepine was converted to amorphous form.

### 2.5. In Vitro Drug Release Profile

All formulations of solid dispersions, except SD_1:1, had faster release rates than pure carbamazepine and commercial immediate-release carbamazepine tablets. The fastest release rate was from SD_1:6, followed by SD_1:4 and SD_1:2. The increase of Neusilin^®^ UFL2 content in formulations resulted in the increase of release rate of carbamazepine from formulations. Improvement of dissolution rate from solid dispersions can be explained by several mechanisms: decreased crystallinity, particle size reduction, improved wettability and porosity of particles in formulations [13].

**Figure 5:**
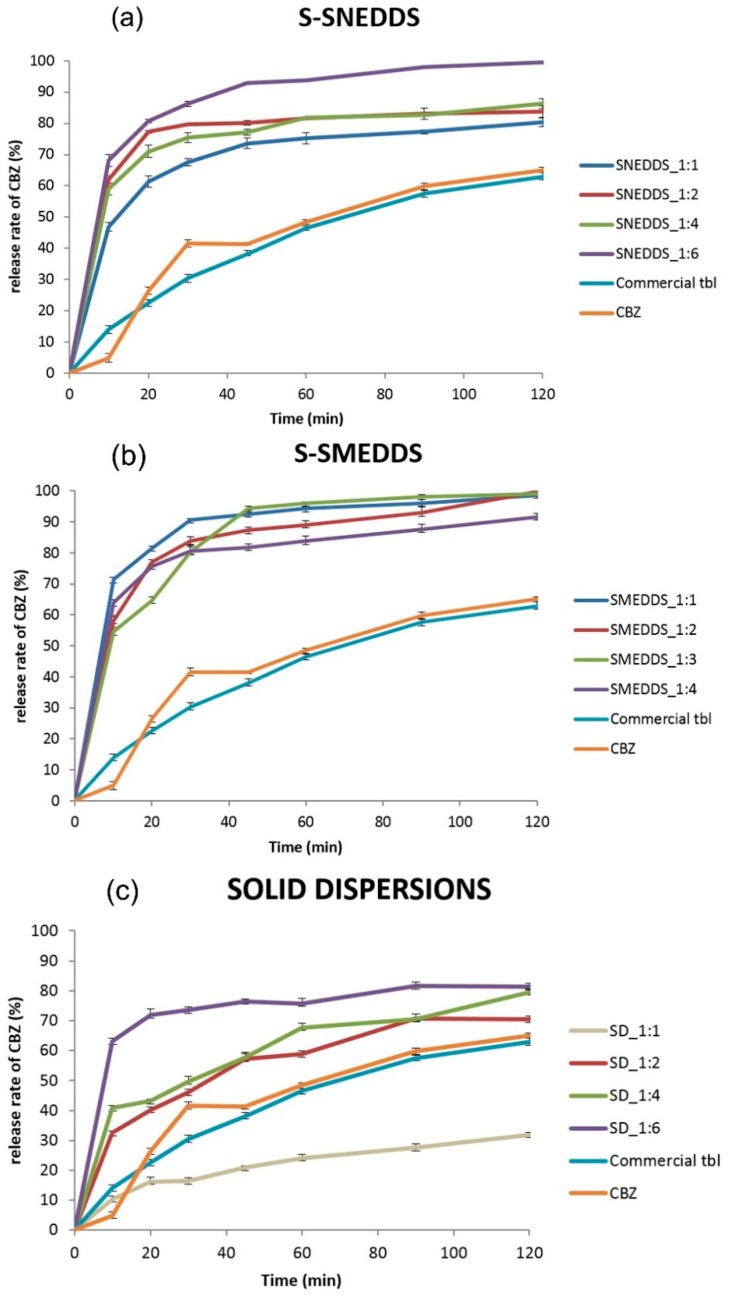
*In vitro* dissolution profiles ofcarbamazepine and commercial tablets in comparison with formulations prepared in our laboratory: S-SNEDDS (**a**); S-SMEDDS (**b**); and solid dispersions (**c**).

All S-SMEDDS formulations showed a significant increase of dissolution rate in comparison with pure carbamazepine and commercial immediate-release carbamazepine tablets (Figure 5). The extent of carbamazepine release from carbamazepine-loaded S-SMEDDS can be explained by a high specific area of adsorbent which adsorbs liquid SMEDDS inside the pores, limiting drug exposure to the surface [27,28]. The presence of SMEDDS most likely enables solubilization of carbamazepine by creating microemulsions. In the first 30 min, the highest dissolution rate was reached from SMEDDS_1:1 (90.63%), followed by SMEDDS_1:2, SMEDDS_1:4 and SMEDDS_1:3 (the release rates from each of these formulations were over 80%). From 45 min onward, the dissolution rate of carbamazepine was slightly higher from the SMEDDS_1:3, compared to other three formulations. A high specific surface area of Neusilin^®^ UFL2 provides better wetting of powder, which increases the release rate of carbamazepine. [29]. Due to a higher ratio of carrier (SMEDDS_1:3 and SMEDDS_1:4), liquid SMEDDS probably becomes entrapped in the long and narrow pores of the carrier, which could cause slower initial release of carbamazepine. Another assumption is that due to the smaller ratio of liquid SMEDDS in formulation, a smaller amount of carbamazepine is solubilized, which can lead to initial slower release.

CBZ dissolution profiles from SMEDDS, calculating difference (f1) and similarity factors (f2), it can be concluded that there is no statistically significant difference between these formulations, since f1 values are between 0 and 15 and f2 values greater than 50 for each comparison.

All S-SNEDDS formulations showed faster release rates in comparison with pure carbamazepine and commercial immediate-release carbamazepine tablets. SNEDDS_1:6 showed the fastest release rate of all SNEEDS formulations (68.17% in the first 10 min, 80.82% after 20 min and 93.01% after 45 min). This could be attributed to a high ratio of Neusilin^®^ UFL2, an adsorbent with a high specific surface (300 m^2^/g). Droplets of SNEEDS with carbamazepine are entrapped in the pores of the adsorbent, which limits drug exposure to the surface of Neusilin^®^ UFL2. A high level of exposure to the surface may result in precipitation of the drug, which decreases the extent of drug release [29]. SNEDDS_1:2 and SNEDDS_1:4 showed a similar drug release profile: 62.01% and 58.92% at 10 min, 77.28% and 71.04% at 20 min, 79.71% and 75.52% at 30 min, respectively and almost identical release rates after 60 min. The lowest release rate of all SNEEDS observed for SNEDDS_1:1 formulation were: 46.83% at 10 min, 61.38% at 20 min, 67.57% at 30 min. From 45 min onward, the dissolution rate was slightly lower in comparison with SNEDDS_1:4. The lowest dissolution rate of SNEDDS_1:1 may be attributed to low adsorbent content in the formulation, compared to other three formulations.

According to the results obtained from dissolution test, all formulations had faster release rates of carbamazepine in comparison with pure carbamazepine and commercial immediate-release carbamazepine tablets. This could be due to the highly specific surface area of Neusilin^®^ UFL2 (300 m^2^/g), as well as carbamazepine–carrier interactions. In comparison with solid dispersions, S-SMEDDS and S-SNEDDS had faster release rates of carbamazepine (50%–70% in the first 10 min), probably due to the influence of two factors: highly specific surface of Neusilin^®^ UFL2 and presence of SMEDDS/SNEDDS. Difference factors (f1) and similarity factors (f2) were calculated in order to compare formulations that had the fastest release rates in comparison with pure carbamazepine and commercial immediate-release carbamazepine tablets. These parameters are presented in Table 1. Two compared formulations are equivalent if f1 values are between 0 and 15 and f2 values greater than 50 (50–100) [24]. According to the results presented in Table 1, all four are significantly different from pure carbamazepine and commercial immediate-release tablets.

**Table 1:** Difference factors (f1) and similarity factors (f2) of formulation and pure carbamazepine and commercial tablets.

		Pure Carbamazepine	Commercial Tablets
**SMEDDS_1:1**	f1	62.17	56.50
	f2	18.49	15.95
**SNEDDS_1:6**	f1	55.33	49.05
	f2	24.40	22.17
**SD_1:6**	f1	54.86	44.02
	f2	24.41	25.62

### 2.6. Parallel Artificial Membrane Permeability Assay (PAMPA)

The results of PAMPA test are given in Table 2. From each formulation type, those that showed the fastest release rate were chosen (SD_1:6, SMEDDS_1:1, SNEDDS_1:6), as well as pure carbamazepine, and then a PAMPA test was performed. The apparent permeability of pure carbamazepine was in accordance with previously reported results for the same drug in a similar PAMPA system [22]. Results of PAMPA test showed that all tested formulations have a higher permeability rate than pure carbamazepine because of the existence of interactions between carbamazepine and carrier.

**Table 2:** PAMPA test: values of apparent permeability coefficients (Papp) of tested formulations and pure carbamazepine.

Formulations	Papp × 10^−6^ (cm/s)
Carbamazepine	11.77 ± 0.34
SD_1:6	16.12 ± 2.27
SNEDDS_1:6	19.12 ± 0.87
SMEDDS_1:1	21.42 ± 1.67

Increase of permeability in SD formulations may be due to particle size reduction and amorphization of the drug. Also, the increase in permeability may be a result of supersaturation from amorphous solid dispersion formulation. As opposed to other solubilization methods, the use of amorphous solid dispersions does not alter the equilibrium solubility of the drug; rather, it enables an unstable supersaturated solution to be attained. Solubilization via surfactants and cosolvents may decrease the apparent membrane permeability. This is because the apparent membrane/aqueous phase partition coefficient, which is a primary determinant of the intestinal membrane permeability, decreases with increasing drug solubility, thereby reducing the thermodynamic driving force for membrane permeation [30]. On the other hand, apparent solubility increased via supersaturation does not result in a decrease in a partition coefficient. This is because the apparent partition coefficient is dictated by the equilibrium solubility of the drug. Since supersaturation has kinetic/nonequilibrium solubility, it will not affect the apparent partition coefficient. Hence, apparent membrane permeability is unaffected by supersaturation while flux (the product of apparent solubility × apparent permeability) increases dramatically. Small structures of amorphous drug/carrier “nanoaggregates” formed in solution play a critical role in maintaining supersaturation by crystallization inhibition [30].

The use of surfactants for solubility increments may also change the permeability in various ways. Surfactants may increase membrane permeability for drugs with inherently low-permeability and high aqueous solubility (*i.e*., BCS class III compounds) through the disruption of membrane integrity to increase paracellular transport. However, for lipophilic drugs with inherently high transcellular membrane permeability (e.g., BCS class II), surfactants can decrease the free fraction of the drug which results in decreased permeability. In using surfactants for the solubilization of lipophilic drugs, it is well recognized that above the critical micelle concentration (CMC), drugs may be incorporated into surfactant micelles. Additionally, it is this micellization process which drives the extraordinary increase in apparent aqueous solubility afforded by surfactants. However, the micellar solubilization also results in decreased free fraction of drug. This incorporation of the drug in micelles significantly decreases the amount of free drug available for permeation through the intestinal membrane. Decreased free fraction is directly translated to lower concentration gradient and hence thermodynamic driving force for membrane permeation [19]. It is reported that SEEDS formulations enhance passive permeability of highly lipophilic drugs, mainly through modulation of membrane fluidity [31]. Among tested formulations, the increase in permeability is especially expressed in formulations SMEDDS_1:1 and SNEDDS_1:6, probably due to interactions within surfactants and artificial membrane, along with carbamazepine–carrier interactions. S-SMEDDS showed the highest permeability rate, which was expected, due to the small particle size of all evaluated formulations.

## 3. Experimental Section

### 3.1. Materials

Cremophor^®^ EL (BASF, Ludwigshafen, Germany), was used as a surfactant in the preparation of SMEDDS. Macrogol 400 was used as a cosurfactant in the preparation of SMEDDS.

Labrasol^®^ (Gattefosse GmbH, Bad Krozingen, Germany), was used as a surfactant in the preparation of SNEDDS. Polysorbate 80 (Tween^®^ 80, Sigma-Aldrich Chemie GmbH, Steinheim, Germany), was used as a surfactant in the preparation of the SNEDDS. Transcutol^®^ HP (Gattefosse GmbH), was used as a cosurfactant in the preparation of the SNEDDS. Mygliol^®^ 812 (Sasol GmbH, Hamburg, Germany), was used as oil in preparation of the SMEDDS and SNEDDS. Neusilin^®^ UFL2 (Fuji Chemical Industry Co., Ltd., Toyama, Japan) was used as a solid carrier for all formulations. CBZ (Ph. Eur. 8.0) was used as a model of poorly soluble active ingredient. Galepsin^®^ (Galenika AD, Belgrade, Serbia) was used as a commercial immediate-release formulation (200 mg). Dodecan (Sigma-Aldrich Chemie GmbH) and egg lecithin (Lipoid GmbH, Ludwigshafen, Germany) were used in the PAMPA test. The water used in all experiments was double-distilled. Absolute ethanol was Ph. Eur. 8.0 grade. All other reagents that were employed for HPLC analysis were of analytical grade.

In this study, several formulation strategies were evaluated as a possible solution to enhance the solubility of carbamazepine: solid dispersions, solid self-microemulsifying drug delivery systems (S-SMEDDS) and solid self-nanoemulsifying drug delivery systems (S-SNEDDS).

### 3.2. Methods

#### 3.2.1. Solid Dispersions

Solid dispersions were prepared by the solvent casting method. Carbamazepine was dissolved in a minimal amount of absolute ethanol and this solution was mixed with Neusilin^®^ UFL2 in a mortar with a pestle. The solvent was then removed by heating at 25 °C in a dry-heater for 24 h. Four different formulations were made in which carbamazepine: Neusilin^®^ UFL2 ratio was varied: 1:1, 1:2, 1:4, 1:6 (marked as SD_1:1, SD_1:2, SD_1:4 and SD_1:6, respectively).

#### 3.2.2. Solid Self-Microemulsifying Drug Delivery Systems (S-SMEDDS)

In these formulations, the surfactant phase was a mixture of Cremophor^®^ EL as the surfactant and Macrogol 400 as the co-surfactant (in a 3:1 ratio). Mygliol^®^ 812 was used as an oil phase. Surfactant phase: the oil phase ratio was 9:1. The ratio of components within SMEDDS was determined in previous screening studies and droplet size and PDI confirmed by photon correlation spectroscopy. SMEDDS was then adsorbed on a mixture of Neusilin^®^ UFL2 as a solid carrier and carbamazepine, previously prepared by thoroughly mixing these two components in a mortar with a pestle. Four formulations were prepared in which the SMEDDS: Neusilin^®^ UFL2 ratio was varied: 1:1, 1:2, 1:3, 1:4 (marked as SMEDDS_1:1, SMEDDS_1:2, SMEDDS_1:3 and SMEDDS_1:4, respectively). Carbamazepine content was constant in all formulations (20%).

#### 3.2.3. Solid Self-Nanoemulsifying Drug Delivery Systems (S-SNEDDS)

The SNEDDS formulation contains 17.29% Mygliol^®^ 812 as oil phase, 46.28% Labrasol^®^/Polisorbate 80 (1:1) as the surfactant, and 36.43% Transcutol^®^ HP as the co-surfactant. The ratio of the components within SNEDDS was determined in previous screening studies and droplet size and PDI confirmed by photon correlation spectroscopy. SNEDDS was then adsorbed on a mixture of Neusilin^®^ UFL2 as a solid carrier and carbamazepine. Four formulations were made, in which the SNEDDS: Neusilin^®^ UFL2 ratio was varied: 1:1, 1:2, 1:4, 1:6 (marked as SNEDDS_1_1, SNEDDS_1_2, SNEDDS_1_3, SNEDDS_1_4, respectively). Carbamazepine content was constant in all formulations (20%).

### 3.3. Photon Correlation Spectroscopy (PCS)

The droplet size of drug-unloaded SMEDDS and SNEDDS was evaluated using photon correlation spectroscopy (PCS) after appropriate dilution with water. PCS measures droplet size by analyzing random intensity fluctuations in light scattering due to Brownian motion of the particles. The intensity correlation function provides information on the translational diffusion coefficient of the scattering particles and hence the hydrodynamics radius according to Stokes-Einstein equation. The droplet size was determined using the apparatus Nano ZS90 (Malvern Instruments, Malvern, UK) equipped with a He–Ne laser at 633 nm at 20 ± 0.2 °C. The size measurements were carried out at a fixed angle of 90°. Droplet size measurements were performed in the samples diluted with the water phase up to 90% *w*/*w*. Computer software performs statistical analysis of data and calculates the average droplet size (Z-Ave) and polydispersity index (PDI) from intensity distribution. The results are the mean and standard deviation (S.D.) of three consecutive measurements for each sample [24,32].

### 3.4. In Vitro Drug Release Studies

Dissolution profiles of SMEDDS, SNEDDS and solid dispersions as well as pure carbamazepine and commercial immediate-release carbamazepine tablets were determined using a rotating paddle apparatus (Erweka DT70, Erweka GmbH, Hausenstamm, Germany). The dissolution conditions were: 37 °C, 900 mL of water (medium), 50 rpm. Samples of 5 mL were withdrawn from the medium at fixed time intervals (10, 20, 30, 45, 60, 90 and 120 min). All samples were filtered and assayed for carbamazepine. Carbamazepine concentration was determined spectrophotometrically at 287 nm (Evolution 300, Thermo Fisher Scientific, Hemel Hempstead, England, UK). The weight of every sample was such that every sample contains 200 mg of carbamazepine.

Release profiles of carbamazepine from prepared formulations were compared with the dissolution profiles of pure carbamazepine and commercial immediate-release carbamazepine tablets by calculating the difference factor (f1) and similarity factor (f2) [24].

### 3.5. Characterization of Selected Formulations

From each type of formulation, the one that showed the fastest release rate of carbamazepine was selected, and then PAMPA test was performed, in order to evaluate the permeability of carbamazepine. Also, characterization of these formulations was performed, using DSC, PXRD and FT-IR spectroscopy.

#### 3.5.1. Fourier-Transform Infrared Spectroscopy (FT-IR)

FT-IR spectra in the region of 600–4000 cm^−1^ for pure carbamazepine and some formulations that are suspected to have an inadequate increase of dissolution rate, were obtained using a Shimadzu IR-Prestige-21 FT-IR spectrometer coupled with a horizontal Golden Gate MKII single-reflection ATR system (Specac, Kent, UK) and equipped with a Zn Se lens.

#### 3.5.2. Differential Scanning Calorimetry (DSC)

DSC analysis were carried out on a computer-interfaced differential scanning calorimeter (DSC Q2000, TA Instruments). DSC was used to determine the presence of polymorphic form of crystalline carbamazepine in samples and also the degree of crystallinity for the encapsulated drug. The instrument was calibrated for temperature and energy using indium standards. The samples were accurately weighed (1–2 mg) and heated from 20–200 °C at a rate of 10 °C/min, under a nitrogen purge gas flow of 50 mL/min. The presence of an endothermic and/or exothermic peak was used as a marker of crystalline form of carbamazepine.

#### 3.5.3. Powder X-ray Diffraction Analysis (PXRD)

Powder X-ray diffraction patterns were measured in order to evaluate crystalline/amorphous character of prepared samples. Measurements were performed using a Bruker AXS D8 Advance powder diffractometer, equipped with copper cathode (λ = 0.15418 nm, 40 kV, 40 mA). Patterns were obtained with step width of 0.02° and a detector in 2θ between 4° and 40° at ambient temperature. Samples, ground into powders with an agate mortar and pestle, were measured on a low background quartz plate in an aluminum holder.

### 3.6. PAMPA Test

The PAMPA test was used to predict passive human gastrointestinal absorption of pure carbamazepine and formulations of each formulation type that had the fastest release rate.

In this test, a hydrophilic polyvinylidene difluoride (PVDF) 96-well filtration plate (Millipore, Bedford, MA, USA) was used as the carrier of the artificial membrane and as the receiving plate. The filter material of each well in the filtration (receiving) plate was coated with 5 μL of egg lecithin solution in dodecane (1%, *w*/*v*). The receiving plate was placed onto the donor plate (Millipore, Bedford, MA, USA), which had previously been filled with 300 μL of donor solutions (100–200 μM solutions of carbamazepine and its formulations in phosphate buffer pH 5.5). Subsequently, 300 μL of the phosphate buffer solution pH 5.5 was added to each well of the receiving plate. The system was covered with a plastic lid to prevent evaporation and was incubated for two hours at room temperature. After incubation, the concentrations of carbamazepine in the corresponding wells of the receiving plate as well as in the starting solutions were determined by use of previously described HPLC method [18]. The apparent permeability coefficients (Papp) were calculated using the following equations:

(1) $$\%T=100\cdot \frac{A_{R}\cdot V_{R}}{A_{D0}\cdot V_{D}}$$

(2) $$P_{app}=\frac{V_{D}\cdot V_{R}}{(V_{D}+V_{R})\cdot S\cdot t}\ln\left[\frac{100\cdot V_{D}}{100\cdot V_{D}-\%T\cdot (V_{D}+V_{R})}\right]$$

*V_D_* and *V_R_* are the volumes of the donor and receiving solutions, respectively (mL); *A_D_*_0_ and *A_R_* are the HPLC peak areas of the initial and receiving solutions, respectively; *S* is the surface area of the artificial membrane (0.28 cm^2^, according to the manufacturer); *t* is the incubation time (s) and %*T* is extent of transport across the membrane [22].

## 4. Conclusions

An increase in carbamazepine dissolution was achieved in all formulations in comparison with pure carbamazepine and commercial immediate-release carbamazepine tablets. S-SMEDDS and S-SNEDDS formulations had higher release rates of carbamazepine solid dispersions. The highest dissolution rate and permeability of carbamazepine was accomplished from SMEDDS_1:1. These formulations weighed approximately 500 mg, and contain 20% carbamazepine, which is far more acceptable than other formulations found in the literature. The impact of solubility enhancement on permeability of carbamazepine was assessed by use of the PAMPA test. It was also demonstrated that the *in vitro* permeability rate of carbamazepine was increased in tested formulations. The results of this work suggest that the proper selection of the ratio of components in solubility-enhancing formulations may be crucial for achieving appropriate bioavailability of poorly soluble drugs. Also, the impact of solubility increase on permeability of the drug must be considered when preparing such formulations, in order to achieve an optimal solubility–permeability balance, and maximize the overall absorption of these drugs.
